# Differential to Terminal Ileitis: Terminal Ileum Neuroendocrine Tumor Identified on Screening Colonoscopy

**DOI:** 10.31486/toj.22.0035

**Published:** 2023

**Authors:** Alp S. Kahveci, Muhammad F. Mubarak, Isma Perveze, Ebubekir S. Daglilar

**Affiliations:** ^1^ School of Medicine, University of Missouri, Columbia, MO; ^2^ Department of Gastroenterology, University of Missouri, Columbia, MO; ^3^ Department of Pathology and Anatomical Sciences, University of Missouri, Columbia, MO

**Keywords:** *Colonoscopy*, *ileal neoplasms*, *ileitis*, *ileum*, *neuroendocrine tumors*

## Abstract

**Background:** Asymptomatic patients at average risk of developing colorectal cancer are encouraged to undergo screening colonoscopy beginning at age 45 years. While ileal intubation is often considered the gold standard for a complete colonoscopy, the relatively low diagnostic yield has prevented widespread adoption. Small bowel cancers, including neuroendocrine tumors, may present incidentally as terminal ileitis on routine colonoscopy with terminal ileum intubation. Neuroendocrine tumors, the most common primary neoplasm of the small intestine, are often asymptomatic or present as nonspecific abdominal pain in the sixth or seventh decade of life.

**Case Report:** A 51-year-old asymptomatic male with unremarkable physical examination underwent screening colonoscopy that revealed scattered ulcerations of the terminal ileum. Immunohistochemistry of the lesion was consistent with well-differentiated neuroendocrine tumor, World Health Organization Grade I. DOTATATE positron emission tomography/computed tomography demonstrated avid adjacent right mesenteric lymph node and avid focal pancreatic body lesion. Fine-needle biopsy and immunohistochemistry of the pancreatic lesion confirmed neuroendocrine tumor, while the mesenteric lymph node was found to be benign. The patient underwent robotic-assisted ileocolic resection and has ongoing surveillance of the pancreatic lesion.

**Conclusion:** Terminal ileitis encompasses a host of pathologic processes, including inflammatory states, infectious disease, malignancy, and vasculitis. Importantly, small bowel cancer is an increasing cause of terminal ileitis. Screening colonoscopy with ileal intubation can be a valuable tool for early detection of these lesions.

## INTRODUCTION

In May 2021, the US Preventive Services Task Force updated the recommendations regarding routine screening for the detection of colorectal cancer in asymptomatic patients at average risk.^1^ Beginning at age 45 years, patients are encouraged to undergo one of several screening options, including an annual fecal immunochemical test, flexible sigmoidoscopy every 5 years, or colonoscopy every 10 years.

Although not required for a screening colonoscopy, ileal intubation is frequently considered as an adjunct for achieving a complete examination.^2^ However, concerns about the added time of the procedure and a diagnostic yield <5% in asymptomatic patients have prevented widespread adoption of ileal intubation.^3,4^

The small intestine composes nearly 90% of the surface area of the gastrointestinal tract, but cancers of the small intestine account for <4% of the neoplasms identified in the entire gastrointestinal tract.^5,6^ We present a case of asymptomatic small intestine neuroendocrine tumor of the terminal ileum that was identified following visualization of terminal ileitis on screening colonoscopy with terminal ileal intubation.

## CASE REPORT

A 51-year-old male with a medical history of hypertension was referred by his primary care provider for screening colonoscopy. The patient was at average risk for colon cancer with no known personal or family history of colorectal cancer. He was a never-smoker and had had no previous surgeries. The patient's physical examination findings on presentation were unremarkable.

**Figure 1.**
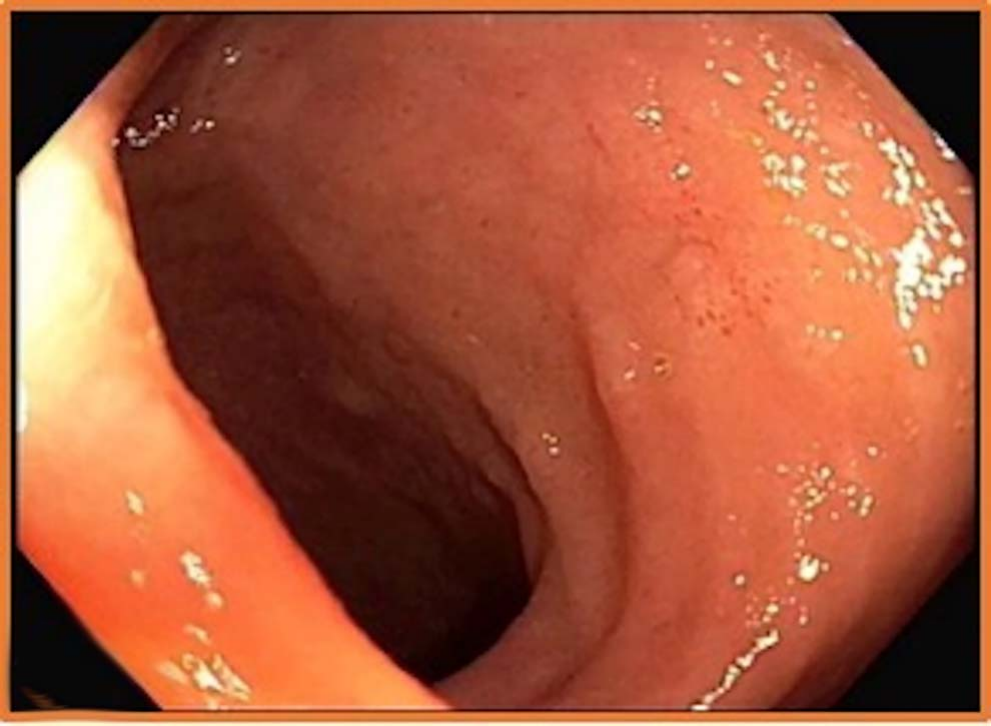
Clean-based ulcerations of terminal ileum seen on screening colonoscopy.

**Figure 2.**
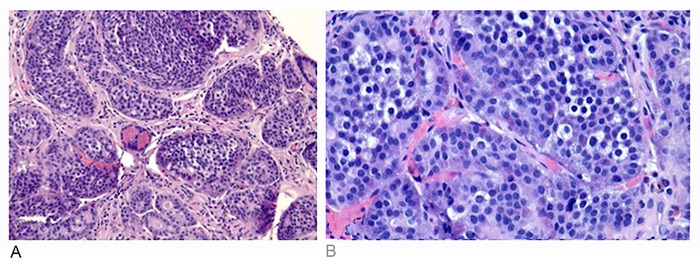
(A) Terminal ileum biopsy revealed uniform cells arranged in organoid architecture with smooth nuclear membranes (magnification ×200). (B) Tumor cells consisted of uniform nuclei, smooth nuclear membranes, and salt and pepper chromatin (magnification ×400).

**Figure 3.**
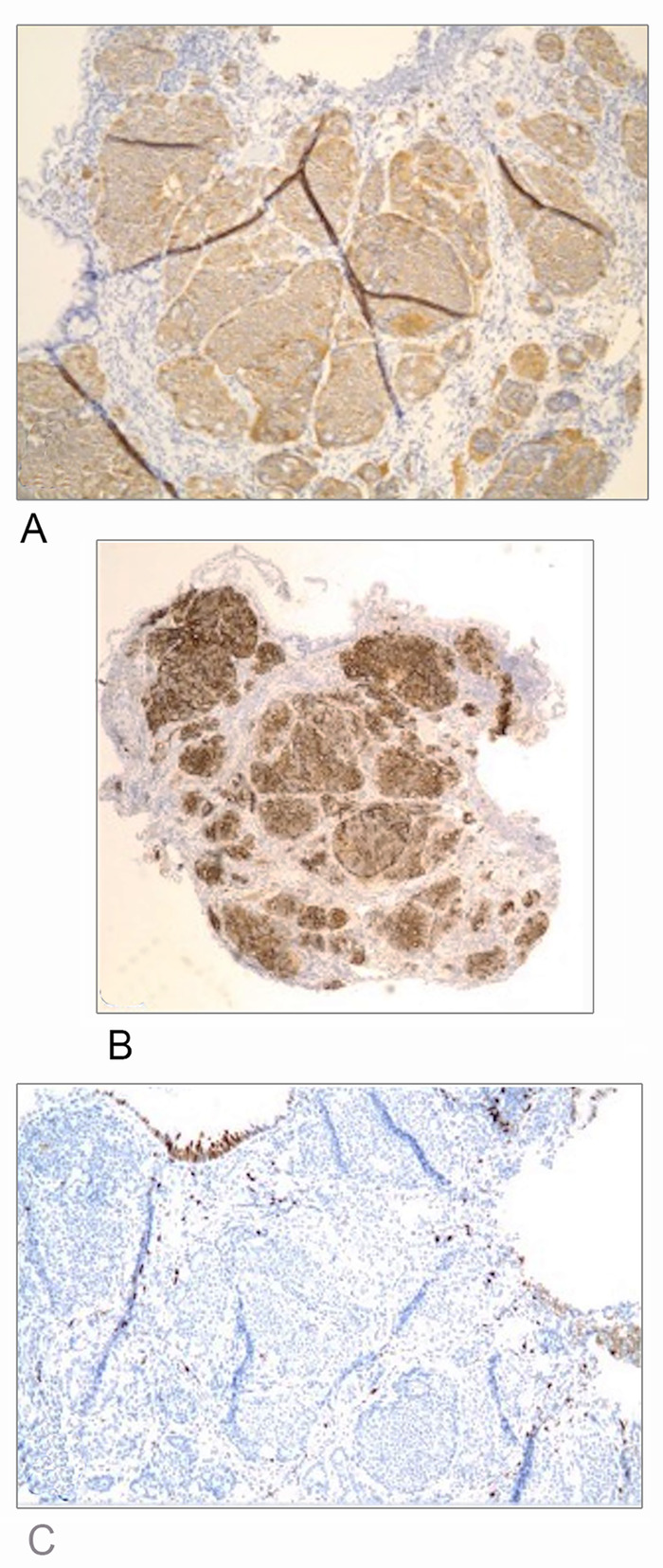
Tumor cells were positive for (A) synaptophysin and (B) CD56, but (C) less than 1% of cells stained positive for Ki-67.

Screening colonoscopy demonstrated scattered, clean-based, 1- to 3-mm ulcerations of the terminal ileum with patchy erythema and edema (Figure 1). Biopsies of the terminal ileum revealed a well-differentiated neuroendocrine tumor, World Health Organization (WHO) Grade I (Figure 2). Tumor cells were positive for synaptophysin, CD56, and cytokeratin AE1/AE3 (Figures 3A and 3B). Ki-67 proliferation index was <1% (Figure 3C). Chromogranin immunostaining was noncontributory (53 ng/mL).

**Figure 4.**
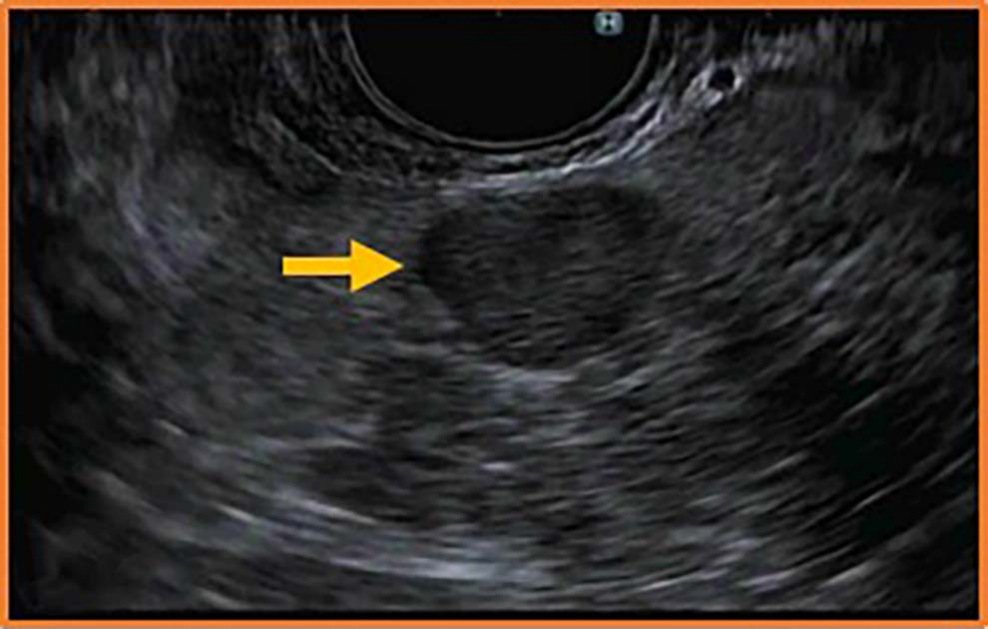
Well-circumscribed hyperechoic lesion of the pancreatic body (arrow) visualized on endoscopic ultrasound.

Staging imaging with computed tomography of the chest, magnetic resonance imaging (MRI) of the abdomen, and DOTATATE positron emission tomography/computed tomography (PET/CT) demonstrated ileocecal junction lesion, avid adjacent right mesenteric lymph node, avid focal pancreatic body lesion, and nonavid enlarged subcarinal lymph node. Because of concerns about regional lymph node involvement and possible distant metastasis, esophagogastroduodenoscopy (EGD), upper endoscopic ultrasound (EUS) with fine-needle biopsy, and bronchoscopy were performed. EGD was unremarkable. However, EUS revealed a focal, well-circumscribed hyperechoic lesion of the pancreatic body measuring 10.1 × 7.7 mm consistent with a primary neuroendocrine tumor (Figure 4). Fine-needle biopsy of the pancreatic lesion confirmed neuroendocrine tumor, while the avid right mesenteric lymph node was found to be benign.

Immunohistochemistry of the pancreatic lesion revealed CD56, synaptophysin, and chromogranin positivity, supporting the diagnosis of neuroendocrine tumor. Bronchoscopy with sampling of a station 7 lymph node was negative for malignant findings.

Because of the presence of multiple neuroendocrine tumors, the patient was referred for genetic testing, but he did not undergo the testing.

The patient underwent robotic-assisted ileocolic resection. Final pathology revealed a stage III, WHO Grade I well-differentiated neuroendocrine tumor with involvement of 6 of 15 lymph nodes. He was discharged home after an uneventful 2-day hospital stay. At 2-week and 3-month follow-up, the patient was doing well with no acute concerns.

The patient's pancreatic neuroendocrine tumor was discussed during multidisciplinary conference with surgical oncology. Given the lesion size <2 cm, the patient was enrolled in a surveillance program. CT scan of the chest, abdomen, and pelvis at the 3-month follow-up appointment revealed no evidence of distant metastases. At the 10-month appointment, the pancreatic lesion was no longer evident on MRI of the abdomen. The patient is set to return to the surgical oncology clinic in 6 months for repeat abdominal MRI and PET/CT.

## DISCUSSION

Terminal ileitis encompasses a host of pathologic processes, including inflammatory states, infectious disease, malignancy, and vasculitis. Most commonly, terminal ileitis is discussed within the context of Crohn disease, an idiopathic transmural inflammation of the gastrointestinal tract.^7,8^ Inflammation is often noncontiguous, manifesting as skip lesions between the mouth and the anus. These skip lesions may progress into ulcers that eventually form fistulas and strictures. Histologically, Crohn disease is characterized by the formation of noncaseating granulomas. In contrast to Crohn disease, ulcerative colitis is restricted to the mucosa and/or submucosa, involves continuous colonic lesions, and is characterized histologically by the formation of crypt abscesses. The development of terminal ileitis in ulcerative colitis is secondary to a weakened ileocecal valve, whereby colonic contents are refluxed into the terminal ileum, a condition called backwash ileitis.^7,8^ Backwash ileitis is estimated to occur in up to 17% of patients with ulcerative colitis.^9^ Ileal erosions can occur but are rare.^8^

Intestinal infections are another important consideration in the differential of terminal ileitis. One such infection is *Mycobacterium tuberculosis*. Although the incidence of tuberculosis has continued to decline in the United States,^10^ the immunocompromised, including patients with HIV and patients receiving biologic therapy, remain at risk for development of intestinal tuberculosis.^8^ Notably, ileocecal involvement has been observed in nearly 90% of patients with intestinal tuberculosis.^11,12^ Ileocecal involvement is likely the result of the high density of lymphoid cells in the terminal ileal area, as well as a favorable growth environment.^7^ Chronic inflammation in the setting of intestinal tuberculosis results in the development of fibrosis and/or stenosis, as well as tuberculomas that can perforate the terminal ileum.^7^ Because intestinal tuberculosis may present similarly to Crohn disease and ulcerative colitis, culture, polymerase chain reaction testing, and biopsy are essential in the diagnostic approach.^7,13^

Another consideration in the workup of terminal ileitis is chronic nonsteroidal anti-inflammatory drug (NSAID) use. Although NSAID use is more commonly associated with peptic ulcer formation, the prevalence of NSAID-induced enteropathy is increasing.^14^ Small bowel injury can vary from mild mucosal inflammation to ulceration. While rarely seen, diaphragmatic strictures are pathognomonic for chronic NSAID use.^15^ Patients are generally asymptomatic, although some present with iron-deficiency anemia.

Medications other than NSAIDs have also been implicated in the development of terminal ileitis. Immune checkpoint inhibitors have been found to cause terminal ileitis.^16^ The withdrawal of the offending drugs results in resolution of the findings. In some cases, treatment with systemic steroids may be required.^17-20^

Although not frequent, small bowel cancer is an increasing cause of terminal ileitis.^7^ Between 1975 and 2018, the incidence of small bowel cancer per 100,000 individuals in the United States increased from 1.1 to 2.4, a 118% increase.^21^ Among these cancers, neuroendocrine tumors are a type of slow-growing malignancy derived from endocrine cells. The most common primary neoplasm of the small intestine, neuroendocrine tumors most frequently present in the sixth or seventh decade of life.^5,22,23^ Since the 1970s, the incidence of small intestine neuroendocrine tumors has been rising, with analysis of the Surveillance, Epidemiology, and End Results registry through 2012 revealing an incidence of 1.05 per 100,000 persons.^24,25^ Notably, the terminal ileum is the most frequently implicated site within the small intestine.^26^

Because of the secretion of neurotransmitters, including serotonin, neurokinin A, and histamine, patients with small intestine neuroendocrine tumors may present with symptoms consistent with carcinoid syndrome such as flushing, diarrhea, valvular heart disease, and bronchospasms.^27,28^ However, as most patients report nonspecific abdominal pain or are asymptomatic, small intestine neuroendocrine tumors are often incidental findings on routine colonoscopy.^24,26^ Approximately 30% of patients will be metastatic at presentation, often to the liver.^24,29^

Small intestine neuroendocrine tumors can be diagnosed with anatomic imaging, functional imaging, biochemical workup, or endoscopic examination. Most commonly, patients undergo a multiphase CT scan.^30^ Although more expensive, MRI offers greater sensitivity than CT in detection of metastases.^31^ Physiologic function is often assessed by PET/CT in conjunction with somatostatin receptor radiotracers Ga-DOTATOC, Ga-DOTANOC, or Ga-DOTATATE.^30,32^ In addition, patients with small intestine neuroendocrine tumors suspected of having carcinoid syndrome may undergo biochemical testing. Among the many biomarkers to choose from, chromogranin A and 5-HIAA are favored for their high sensitivity and specificity.^27,28,33^

Pathologic examination is necessary to confirm the initial diagnosis of small intestine neuroendocrine tumor. Besides histology, immunohistochemistry with chromogranin and synaptophysin is frequently performed.^34,35^ To determine the appropriate WHO classification of the small intestine neuroendocrine tumor, the Ki-67 proliferative index and mitotic rate are quantified.^36^

The preferred management of locoregional small intestine neuroendocrine tumors is surgical resection, with segmental small bowel resection or ileocecectomy with regional mesenteric lymphadenectomy.^37^ Notably, a retrospective study of well-differentiated small intestine neuroendocrine tumors treated with surgical resection found a 42% recurrence rate, with liver metastases identified in 64% of those who relapsed.^38^ In cases of metastases, somatostatin analogs are preferred as the first-line management because of their antiproliferative effects and ability to suppress carcinoid symptoms.^39,40^

Given the slow rate of regrowth of small intestine neuroendocrine tumors, asymptomatic patients are initially closely monitored with radiographic imaging (eg, every 3 to 4 months), transitioned to 6-month surveillance appointments, and then followed annually with imaging and clinical and biochemical workup for 10 years.^24,40^ The prognosis for small intestine neuroendocrine tumor remains favorable, with a median survival of 103 months for patients with moderately and well-differentiated tumors and a 5-year survival rate of 69%.^25^

## CONCLUSION

This case underscores the importance of screening colonoscopy with ileal intubation in the detection of asymptomatic neuroendocrine tumors of the small intestine. Furthermore, this case serves as a reminder that such neoplasms, although rare, should remain on the differential when considering lesions of the terminal ileum.

## Notes

*This article meets the Accreditation Council for Graduate Medical Education and the American Board of Medical Specialties Maintenance of Certification competencies for Patient Care and Medical Knowledge.*
